# DNA Methylation Profiling of Embryonic Stem Cell Differentiation into the Three Germ Layers

**DOI:** 10.1371/journal.pone.0026052

**Published:** 2011-10-07

**Authors:** Takayuki Isagawa, Genta Nagae, Nobuaki Shiraki, Takanori Fujita, Noriko Sato, Shumpei Ishikawa, Shoen Kume, Hiroyuki Aburatani

**Affiliations:** 1 Genome Science Division, Research Center for Advanced Science and Technology, University of Tokyo, Tokyo, Japan; 2 Divisions of Stem Cell Biology, Institute of Molecular Embryology and Genetics, Kumamoto University, Kumamoto, Japan; 3 Department of Allergy and Immunology, Tokyo Metropolitan Institute of Medical Science, Tokyo, Japan; 4 Department of Human Pathology, Graduate School of Medicine, The University of Tokyo, Tokyo, Japan; Ludwig-Maximilians-Universität München, Germany

## Abstract

Embryogenesis is tightly regulated by multiple levels of epigenetic regulation such as DNA methylation, histone modification, and chromatin remodeling. DNA methylation patterns are erased in primordial germ cells and in the interval immediately following fertilization. Subsequent developmental reprogramming occurs by *de novo* methylation and demethylation. Variance in DNA methylation patterns between different cell types is not well understood. Here, using methylated DNA immunoprecipitation and tiling array technology, we have comprehensively analyzed DNA methylation patterns at proximal promoter regions in mouse embryonic stem (ES) cells, ES cell-derived early germ layers (ectoderm, endoderm and mesoderm) and four adult tissues (brain, liver, skeletal muscle and sperm). Most of the methylated regions are methylated across all three germ layers and in the three adult somatic tissues. This commonly methylated gene set is enriched in germ cell-associated genes that are generally transcriptionally inactive in somatic cells. We also compared DNA methylation patterns by global mapping of histone H3 lysine 4/27 trimethylation, and found that gain of DNA methylation correlates with loss of histone H3 lysine 4 trimethylation. Our combined findings indicate that differentiation of ES cells into the three germ layers is accompanied by an increased number of commonly methylated DNA regions and that these tissue-specific alterations in methylation occur for only a small number of genes. DNA methylation at the proximal promoter regions of commonly methylated genes thus appears to be an irreversible mark which functions to fix somatic lineage by repressing the transcription of germ cell-specific genes.

## Introduction

During embryonic development, different cell types arise in the body through activation of tissue-specific gene expression. This specification is regulated by epigenetic mechanisms such as histone or DNA modification, which can modulate chromatin architecture. This epigenetic machinery stabilizes the expression of cell type-specific genes and represses genes essential for cell fate decision of unrelated lineages or for maintenance of pluripotency [1].

The regulation of developmental genes through histone modification has been well documented, but the role of DNA methylation in such regulation is unclear. It has been shown that DNA methylation is essential for embryogenesis; DNA methyltransferase (Dnmt1)- or Dnmt3b-deficient mouse embryos die before embryonic day 10.5 and, although Dnmt3a-deficient mice occasionally reach term, they suffer serious malformations and die within weeks of birth [2], [3].

DNA methylation at CpG dinucleotides is considered a key mechanism of transcriptional regulation [4], [5], and is involved, for example, in X chromosome inactivation, transposon inactivation and genome imprinting [6], [7]. These studies indicate that DNA methylation functions as a stable silencing mark in heterochromatin formation [1], [8], [9].

It has been widely assumed that promoters in ES cells lack DNA methylation, based on the fact that ES cells are derived from blastocysts after a global demethylation event following fertilization[10], [11], [12]. It was therefore proposed that DNA methylation might be involved in the maintenance of tissue-specific gene expression during differentiation [13], [14], [15]. Although the role of DNA methylation during tissue differentiation in early development remains poorly characterized, recent technological advances [16], [17], [18] are now beginning to reveal global patterns of DNA methylation in tissues. *In vitro* differentiation of mouse ES cells provides an opportunity to study methylation during the epigenomic transition along with cellular differentiation. We used an *in vitro* differentiation system to compare DNA methylation profiles among the three germ layers (ectoderm, endoderm, and mesoderm). This system allowed us to trace genome-wide DNA methylation patterns during the lineage commitment of ES cells, and to compare these patterns across the three germ layers and adult tissues. This study presents a comprehensive map of promoter DNA methylation during lineage commitment in ES cells after segregation into the three germ layers.

## Materials and Methods

### Cell lines, differentiation of ES cells, primary tissues, and sample preparation

The male ES cell line, SK7 [19], [20] containing a Pdx1 promoter-driven GFP reporter transgene expresses undifferentiated ES cell-specific markers such as Oct 3/4, Nanog, SSEA-1 and E-cadherin [20]. Karyotype analysis of SK7 shows normal murine diploid chromosomes with no apparent abnormalities [20]. SK7 ES cells were differentiated into the three germ layers as previously described [21]. The ES cell line, R1, provided by Dr. Andras Nagy (Toronto University) was maintained on MEF feeder cells in Dulbecco's modified Eagle's medium (DMEM; Invitrogen, Carlsbad, CA) supplemented with leukemia inhibitory factor (LIF), 10% FBS, nonessential amino acids (NEAA), L-glutamine (L-Glu), penicillin and streptomycin (PS), and β-mercaptoethanol (β-ME) as previously described [20]. ICR mice were purchased from the Oriental Yeast, Tokyo, Japan. Primary tissues were isolated from male ICR (CD-1) mice that were more than nine weeks old. Genomic DNA was extracted using the QIAamp DNA Micro kit (QIAGEN, Hilden, Germany). RNA was isolated using TRIzol (Invitrogen) according to the manufacturer's instructions. All experiments using mice received approval from the University of Tokyo.

### Expression profile analysis

RNA expression data were analyzed using a Gene Chip Mouse Genome 430 2.0 array (Affymetrix, Santa Clara, CA, USA) containing probes for approximately 39,000 mouse transcripts. Testis expression data was obtained from previous publication's data (GSM127093)[22]. For global normalization, the average signal in an array was designated as 100.

### Methylation profiling by methylated DNA immunoprecipitation (MeDIP)

A MeDIP assay was performed using 2 µg of fragmented DNA (200–700 bp) as previously described [16], [23]. Immunoprecipitation was repeated twice. Immunoprecipitated DNA (IP DNA) and 30 ng of input DNA were amplified by *in vitro* transcription (IVT) as described [24], and hybridized to a GeneChip Mouse Promoter 1.0R array (Affymetrix, Santa Clara, CA, USA) according to the manufacturer's instructions. All MeDIP assays were performed with replicates.

### Bisulfite treatment, bisulfite sequencing, and mass spectrometry measurements

Genomic DNA (1 µg) was fragmented by sonication, and bisulfite treatment was performed as described previously [25]. Mass spectrometry measurements were performed using a MassARRAY mass spectrometer (SEQUENOM, Inc.) according to the manufacturer's instructions. Spectra were analyzed using proprietary peak picking and spectra interpretation tools. PCR assays were performed using the primers listed in Table S1. PCR conditions were: 95°C for 3 min followed by 40 cycles of 95°C for 30 sec, 52°C for 30 sec and 72°C for 1 min.

### Calculation of probe CpG content

We determined the CpG content of the probes to count the number of CpG dinucleotides in 500-bp windows centered on the probe.

### Bioinformatics analysis

Methylation data was compared with genomic features obtained from the UCSC genome browser (*Mus musculus* NCBI Build 36). Initially, in order to define analytic regions, we used regions with a model based analysis of tiling-array (MAT)score greater than 2.5 in at least one sample as candidate methylated regions (CMR) for further analysis, and combined overlapping regions among samples into a single region. The MATscore of analytic regions with a CpG density above 5% was then estimated. A MATscore of 3.0 (false discovery rate (FDR): 4.05%) was used to identify the methylated regions with high confidence in at least one sample. Finally, a MATscore of 2.5 was used as a cutoff value in regions in which the MATscore was above 3.0 in at least one sample.

Methylation frequency relative to the distance to the transcription start site (TSS) was calculated using the following formula: (number of probes with a MATscore>2.5/total number of probes spanning the relative distance to the TSS). For sample comparisons, we defined probes that gained methylation in each sample as follows: We first identified regions with a MATscore>3 in at least one sample in a particular region and with a CpG content above 5% in CMR. Within these regions, we then defined methylated regions as regions that fulfilled a MATscore above 2.5 (FDR: 5.81%) and hypomethylated regions as regions that fulfilled a MATscore of less than 1.5 (FDR: 17.49%). We were unable to judge the methylated status of regions with a MATscore between 1.5 and 2.5.

ChIP-Seq data sets profiling the genomic occupancy of Histone lysine27 tri-methylation (H3K27me3) and Histone H3 lysine4 tri-methylation (H3K4me3) in ES cells, neural precursor cells (NPCs) and brain were obtained from previous publications [15], [18] and were re-analyzed using the methods described below. Sequence reads were aligned to NCBI Build 36 (UCSC mm8) of the mouse genome, using ELAND software. Two mismatch errors were allowed for the data sets with 26-bp reads. Only uniquely aligned reads were used for the following analysis. Genomic regions with a specific chromatin mark were identified based on their enrichment for reads at FDR<10^−^4 using the FindPeaks software [26]. We combined these regions with Refseq genes by overlapping with the regions from 2 kb upstream to 2 kb downstream of the TSS. All analytic promoters of Refseq genes were classified as either high CpG density promoters (HCPs), intermediate CpG density promoters (ICPs) or low CpG density promoters (LCPs) according to a previous report [27]. DNA methylation levels of Refseq genes were extracted from the core promoter regions (from 1.5 kb upstream to 0.5 kb downstream of the TSS).

### Gene Set Enrichment Analysis (GSEA)

GSEA was performed using a publicly available desktop application from the Broad Institute (http://www.broad.mit.edu/gsea/software/software_index.html). P-values were calculated by permuting the genes 1000 times. Ranked gene lists were sorted using a testis specific expression score that was calculated using the following formula: (gene chip score of the testis/the maximum gene chip score of somatic cells and tissues (ectoderm, endoderm, paraxial mesoderm, brain, liver, and skeletal muscle)). A set of genes with methylation in common across all somatic samples was created by the following definition: MATscore>2.5 in all somatic samples and MATscore>3 in at least one sample in a region that fulfilled CpG content above 5% in CMRs.

### Accession codes

Microarray data have been deposited in NCBI's the Gene Expression Omnibus and are accessible through GEO Series accession number GSE32082.

## Results

### Genome-wide profiling of promoter DNA methylation in ES cells and in the three ES cell-derived early germ layers

**Figure 1:**
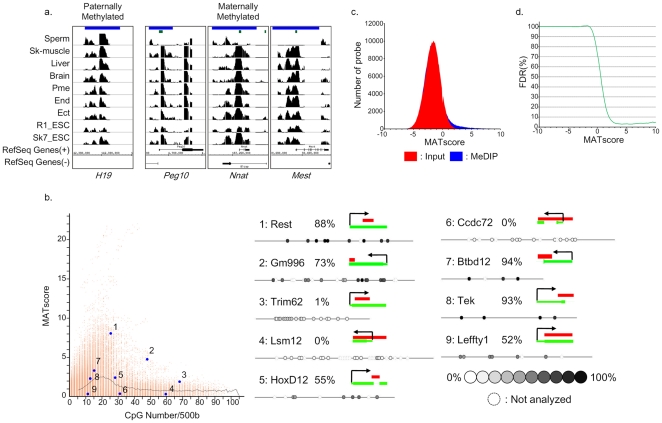
Defining the promoter methylome. a) Microarray detection of DNA hypermethylation on imprinting center regions (ICRs). A green line indicates a CpG island region. A blue line indicates an ICR. b) (left panel) Scatter plots show the DNA methylation levels for all probes relative to their CpG content (CpG/bp). Each spot represents one probe. (right panel) Quantification of DNA methylation for a subset of probes (left panel)**.** The red line indicates the region amplified for bisulfite sequencing. CpGs are represented as open dots (if unmethylated) or filled dots (if methylated). The percentage of CpG methylation is indicated for each amplicon. c) The MATscore distribution of array regions corresponding to INPUT (red) and MeDIP (blue). d) The FDR (%) distribution corresponding to each MATscore.

The role of DNA methylation in ES cell differentiation is not clear. To gain insights into DNA methylation alterations during differentiation of ES cells, we created genome-wide promoter DNA methylation maps of ES cells and of the three ES cell-derived germ layers [21]. The three germ layer lineages derived from ES cells were confirmed by the expression of specific marker genes in each germ layer (Fig. S1.). Using the MeDIP on chip protocol previously described [16], we immunoprecipitated methylated DNA from R1 and SK7 ES cells, as well as fromSK7 derived-ectoderm, -endoderm, -paraxial mesoderm, brain, liver, skeletal muscle, and sperm, and hybridized this DNA to a genome tiling array. The chosen array represents more than 28,000 mouse promoter regions, each covered by 25 oligonucleotides that spanned from 6 kb upstream to 2.5 kb downstream of the TSS. Duplicate tiling array output data (MeDIP1 and MeDIP2 versus Control1 and Control2, for each sample) were analyzed using the Model-based analysis of Tilling array (MAT) program [28], [29]. A number of imprinted gene loci, which were previously reported to have allele-specific methylation, were clearly recognized as highly methylated regions (Fig. 1a).

### Differential methylation of probes relative to CpG density

The DNA methylation levels in ectoderm for all probes relative to their CpG content are shown in Fig. 1b. There was an increase in the average MATscore when CpG content was between 0–4% (CpG<20/500), but a decrease in the MATscore for CpG content between 4–6% and a flat line above 6% (Fig. 1b). This distribution reflects the generally hypomethylated state of CpG-rich probes (above 5% CpG content), indicating that relatively CpG-poor promoters might become methylated in normal tissues. This pattern (Fig. 1b) is consistent with recent findings using different platforms [27], [30], which showed that genes with very low CpG content promoters are constitutively methylated whereas genes with high CpG content are mostly unmethylated. To identify additional CpG methylation within CpG-rich promoters during cellular differentiation, we focused on regions with a CpG content above 5% in subsequent analyses.

To determine a cut off value to define highly methylated regions, we counted the number of probes relative to each MATscore. Fig. 1c shows the distribution of the MATscore of probes in either input or MeDIP, and Fig. 1d shows the FDR (%) relative to each MATscore. These results show that regions with a MATscore greater than 3.0 are methylated regions with high confidence (FDR: 406%). Next, in order to verify the DNA methylation status of regions with various MATscores, we quantitatively analyzed the methylation patterns using a MassARRAY mass spectrometer. Regions with a MATscore above 3.0 showed significant methylation. While most regions (9 of 13 regions) with a MATscore between 2.5 (FDR: 5.81%) and 3.0 showed significant methylation, some of these regions (4 of 13 regions) displayed a hypomethylated status (Fig. S2). We therefore used a MATscore of 3.0 as the cutoff value to determine the presence of significantly methylated regions in at least one sample. In regions with a MATscore above 3.0 in at least one sample, we lowered the cutoff value to a MATscore of 2.5.

### Distribution of DNA Methylation

The total number of CMRs in each sample is summarized in Table 1. In agreement with the results of recent reports, sperm as well as ES cells were more hypomethylated than somatic lineage cells and tissues [30]. Although CpG islands are mostly hypomethylated, they occasionally become heavily methylated, which invariably correlates with silencing of any promoter within the CpG island. DNA methylation of CpG island promoters has been reported to repress transcription when these promoter constructs are introduced into cells [31]. We therefore determined whether CpG islands are methylated. In agreement with previous studies, most CpG islands were hypomethylated, and only a small fraction of CpG islands were hypermethylated (Table 2). Furthermore, hypermethylation of CpG islands increased after differentiation, suggesting that differentiation stimuli induce an increase in DNA methylation levels.

**Table 1:** The number of CMR (Candidate of Methylated Region).

	MATscore>2.5	MATscore>3.0	MATscore>4.0
Sk7_ESC	540	403	279
R1_ESC	361	151	77
Ect	1556	1178	810
End	2149	1513	1052
Pme	2610	1594	948
Brain	1063	849	627
Liver	1127	914	698
Sk_muscle	1377	1071	840
Sperm	405	293	212

**Table 2:** DNA methylation frequency in CpG island.

	Number of MeCpGI			Frequency (%)		
	MATscore>2.5	MATscore>3.0	MATscore>4.0	MATscore>2.5	MATscore>3.0	MATscore>4.0
SK7_ESC	308	213	143	2.49	1.72	1.16
R1_ESC	200	69	40	1.62	0.56	0.32
Ect	946	714	470	7.65	5.78	3.80
End	1332	980	640	10.78	7.93	5.18
Pme	1634	1084	590	13.22	8.77	4.77
Brain	639	498	351	5.17	4.03	2.84
Liver	676	553	406	5.47	4.47	3.29
SK_muscle	814	642	499	6.59	5.20	4.04
Sperm	233	165	126	1.89	1.34	1.02

**Figure 2:**
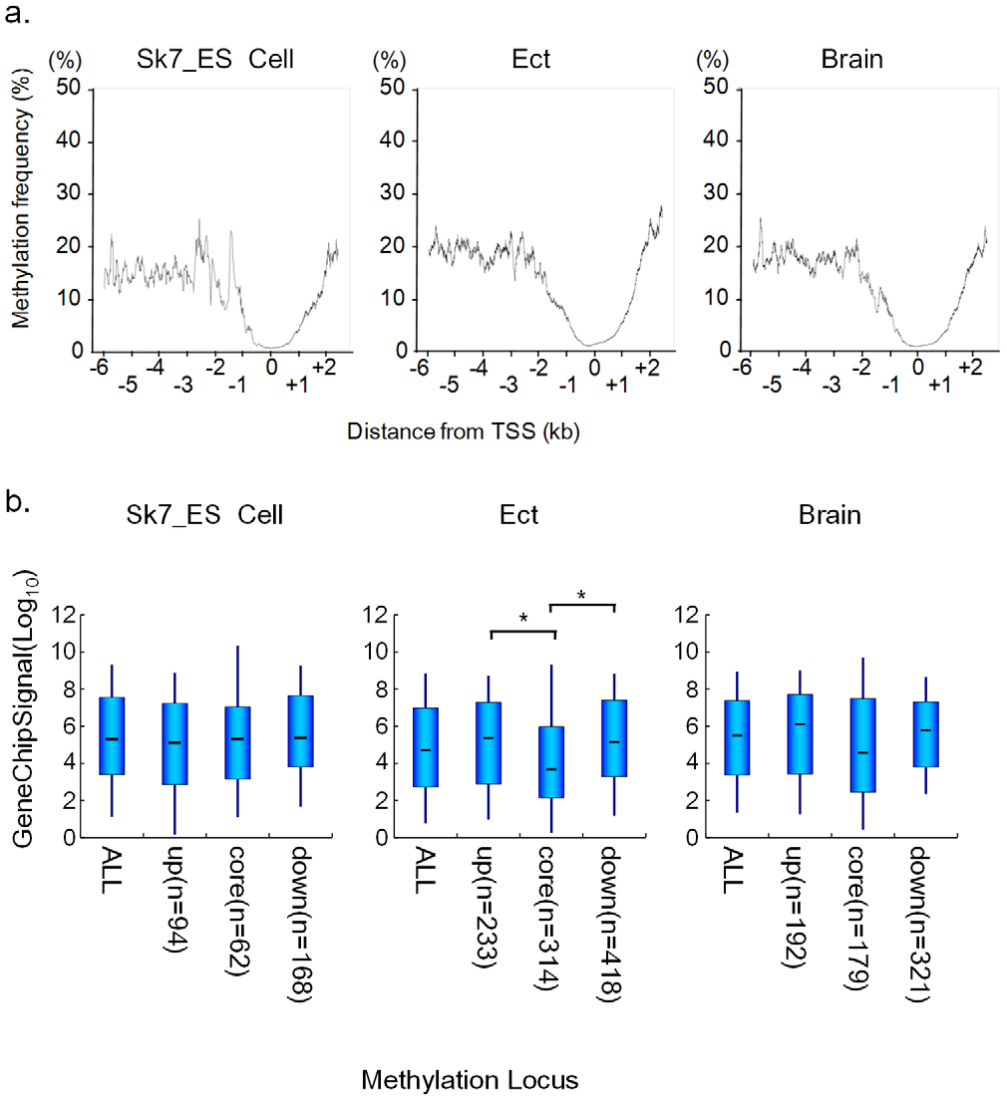
DNA methylation at transcription start sites (TSSs). a) DNA methylation frequency relative to the distance to the TSS. Methylation frequency was calculated using the following formula: Number of methylated probes/Number of total probes at each position from the TSS. The line indicates the moving average. b) Expression levels of genes associated with CMRs in each region around the TSS. The core region shows the region between 1 kb upstream and 0.5 kb downstream of the TSS. Up, indicates 1 kb<upstream from the TSS; down, indicates 0.5 kb<downstream from the TSS. The bold black lines denote medians; boxes denote interquartile ranges, and whiskers denote the 10th and 90th percentiles. (n, number of CMR associated genes) *: pair-wise comparisons of expression levels are significant (*P*<0.01, a *t*-test.).

Next, to analyze the distribution of DNA methylation in promoter regions, we plotted the frequency of DNA methylation relative to the distance from the TSS. DNA methylation levels just around the TSS (±1 kb) were extremely low (Fig. 2a). This hypomethylated status of core promoter regions is consistent with previous reports [32]. However, small fractions of core promoters were hypermethylated (Table 3). To examine the relationship between the distribution of DNA methylation and gene expression, we compared the expression levels of methylated genes in each region in ES cells, ectoderm, and brain. Fig. 2b shows that genes methylated in the core promoter region showed relatively low levels of gene expression compared with genes methylated in other regions. These results indicate that DNA methylation at core promoter regions is associated with gene expression, although most core promoter regions were hypomethylated in all samples.

**Table 3:** The distribution of CMR.

	Total	tss_up	Core (up1k to down 0.5k from tss)	tss_down
SK7_ESC	364	117	62	185
R1_ESC	140	57	13	70
Ect	845	237	236	372
End	913	248	270	395
Pme	920	246	285	389
Brain	740	224	176	340
Liver	756	226	179	351
Sk_muscle	796	239	189	368
Sperm	229	71	25	133

tss_up: >1 kbp upstream from nearest TSS, tss_down: >0.5 kbp downstream from nearest TSS.

### Minimal changes in DNA methylation between the three germ layers

**Figure 3:**
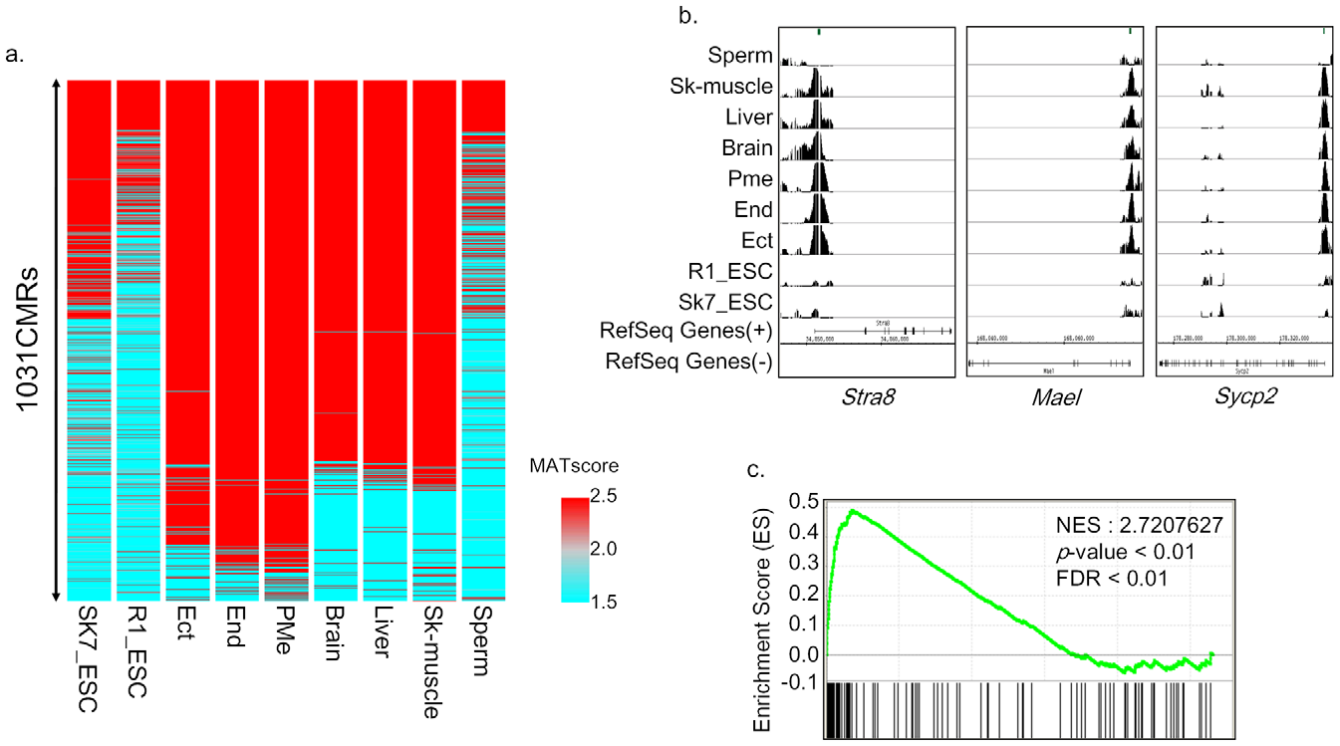
Testis specific genes are commonly methylated in somatic lineages. a) Overview of DNA methylation profiling b) Methylation of germline-specific genes in somatic lineages. A green line indicates a CpG island region. c) Gene Set Enrichment Analysis (GSEA) determines whether a defined set of genes shows concordant changes between two biological states. The normalized enrichment score (NES) reflects the degree to which a gene set is upregulated (positive NES). Corresponding *p* values are indicated.

To identify tissue-specific methylated regions (T-DMR), we extracted 2158 CMRs as methylated regions (MATscore>3) in at least one sample. In these extracted regions, we defined the DNA methylation rate in terms of the MATscore. Thus, a MATscore of less than 1.5 represented a hypomethylated status, and a MATscore greater than 2.5 represented a hypermethylated status (Fig. S2). To detect differentially methylated regions, we extracted and compared hypermethylated (MATscore>2.5) or hypomethylated (MATscore<1.5) regions across somatic samples. Using these criteria, we identified 1031 CMRs. An overview of these 1031 CMRs shows that significant numbers of CMRs were common to all samples, including sperm (Fig. 3a). CMRs that were common to all samples were found in 99 regions, but only 10 of these regions contained CMRs that were located in core promoter regions (Table 4). In contrast, CMRs that were common to somatic samples (the three ES-derived germ layers and adult somatic tissues) were found in 751 regions, and 172 of these CMRs were located in core promoter regions (Table 4). To understand the relationship between common DNA methylation and the expression level of proximal genes, we performed gene ontology analysis of the 172 commonly methylated genes which have methylation in common during early development from the ES cell stage. We found that these common CMRs located in the core promoter region are classified as germ line-specific genes (Table S2 and Fig. 3b). We found that 64 regions were methylated only in the three ES-derived germ layers. However, when we referred these CMRs to a gene ontology database, these CMRs could not be classified into any particular category (data not shown). It was recently reported that human ES cells and human ES-derived definitive endoderm have a larger fraction of methylated regions than do the *in vivo* fetal and adult tissues [14]. Our result using an *in vitro* system of the differentiation of mouse ES cells into the three germ layers (ectoderm, endoderm and mesoderm) is consistent with this report. Collectively, these germ-layer culture-specific methylations are attributed to *in vitro* culture conditions and derivation strategies.

**Table 4:** Distribution of commonly methylated regions around TSS.

	Total	Core	Not core
Total	1031	337	694
All (+)	99	10	89
Sperm (−/+) & Other Samples (+)	186	25	161
Somatic Tissues (+)	751	172	579
Early Diff (+), Adult Tissues (−)	102	64	38

In our study, we detected many methylated regions in ES cells; 421 CMRs in SK7 cells and 199 CMRs in R1 ES cells. We detected 110 regions that were differentially methylated (MATscore<1.5 or>2.5) in SK7 and R1 ES cells as shown in Fig. 3a. The most striking feature is that 95% (104 regions) of the differences between the two ES cell lines are differences where SK7 cells are methylated and R1ES cells are not. However, 80% (83 regions) of the SK7-specific methylated regions were located in non-core promoter regions, and these 110 SK7-specific methylated regions were not associated with the expression level of a proximal gene (data not shown). Brunner and colleagues similarly studied differences in DNA methylation status between two different ES cell lines. They also reported that differences in DNA methylation status were not associated with the expression level of proximal genes. It is known that ESCs are not a uniform group of self-renewing cells but that they shift between inner cell mass (ICM)- and epiblast-like states while retaining pluripotency. Furthermore, the DNA methylation status is different in these two states [33]. This study suggests that the differences in DNA methylation between the SK7 and R1 ES cell lines are the consequence of culture conditions and ES cell heterogeneity.

**Figure 4:**
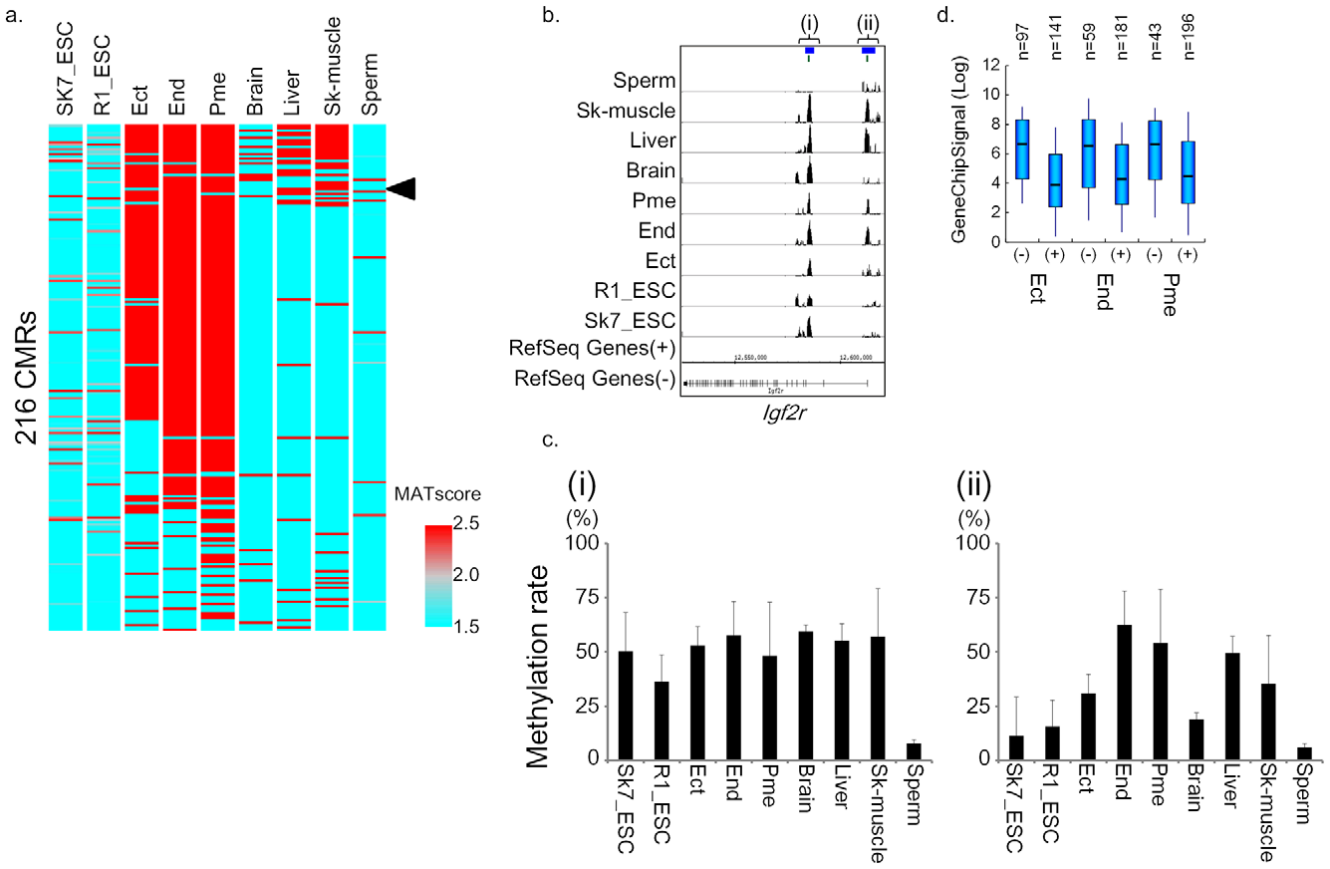
Methylation changes in cells derived from mouse ES cells and mouse adult tissues. a) Overview of variable DNA methylation profiles among the three germ layers. The arrowhead indicates ectodermal relaxation of DNA methylation of *Igf2r*. b) DNA methylation profile and schematic representation of the *Igf2r* imprinting region. A green line indicates a CpG island region. A blue line indicates an imprinting center region (ICR). c) Validation of DNA methylation in the *Igf2r* imprinting region. (i) indicates the normal imprinting region, and (ii) indicates the ectodermal relaxation of DNA methylation. The DNA methylation level was quantitatively estimated using MALDI/TOFMS. d) Expression levels of variable methylation-associated genes for each sample. Pair-wise comparisons of expression levels of methylated or hypomethylated genes were performed using a *t*-test.

We speculated that germ cell-specific genes are commonly methylated across all somatic lineages derived from ES cells. GSEA [34] (Fig. 3c) showed that 172 commonly methylated genes are significantly enriched in testis-specific expressing genes. Notably, the majority of germ line-specific genes we analyzed also showed hypermethylation in somatic cells, but were unmethylated in mature sperm. This result suggests that hypermethylation of germ line-specific genes is involved in the cell fate decision for somatic lineages. This observation is consistent with previous reports that testis-specific promoters are silenced in various somatic tissues and cells [35], [36], [37]. On the other hand, a group of 216 CMRs was differentially methylated in different germ layers (Fig. 4a). Among these genes with differential DNA methylation, we identified the insulin-like growth factor receptor gene (*Igf2r*) (Fig. 4a, b, c), which is known as a tissue-specific imprinted gene and was previously shown to display a tissue-specific promoter relaxation [38]. The mouse *Igf2r* gene and its antisense transcript *Aire* are reciprocally imprinted in most tissues, except for neural tissues where *Igf2r* is biallelicly expressed despite the imprinted *Aire* expression [38]. Some CMRs in these germ layer-specific methylated genes were inversely associated with an elevated expression level of proximal genes (Fig. S3). Therefore, to examine whether this germ layer-specific methylation was related to the expression level of proximal genes, we compared the expression level of methylated or unmethylated genes. Fig. 4d shows that these CMRs were not associated with the expression level of proximal genes.

We next determined how many early T-DMRs were maintained in mature tissues. However, most of the T-DMRs in the three germ layers were not observed in mature tissues and we found only 10 variable CMRs that were present in early differentiation and that sustained a methylated pattern in mature tissues (data not shown). These results may suggest that *de novo* DNA methylation in high CpG content regions has little impact on the establishment of germ layers from ES cells and on tissue diversity.

### DNA methylation in gene cluster regions

**Figure 5:**
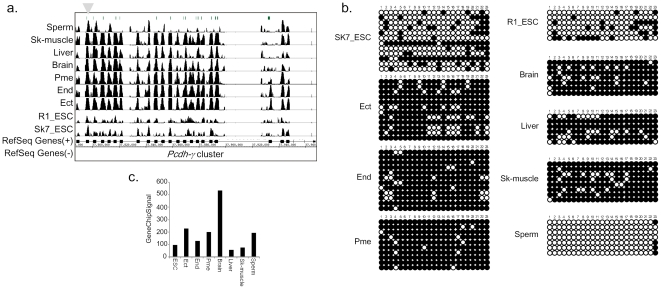
The *Pcdh* cluster is methylated during differentiation into the three germ layers. a) Overview of the DNA methylation profile of the *Pcdh-γ cluster*. A green line indicates a CpG island region. The arrowhead indicates the region analyzed for DNA methylation. b) Bisulfite sequencing analysis of the DNA methylation status of the first exon of *Pcdh-γα2.* c) Gene expression pattern of *Pcdh-γα2*.

Some of the common CMRs were significantly enriched in two specific chromosomal loci. One locus was the 18c region of chromosome 18, and the other locus was the A3.1 region of chromosome X. These two loci contained cluster-type genes. The former locus contains the protocadherin gene family and the latter locus contains the reproductive homeobox X-linked (*Rhox*) gene. Thus, not only germ cell-specific genes but also homophilic cell adhesion genes are enriched in commonly methylated genes. The protocadherin gene family includes three gene clusters, *Pcdh*-α, -β and -γ. Each cluster contains a large region of tandemly arranged and variable exons. In *Pcdh*-α (and -γ (clusters, only one variable first exon is spliced onto constant region exons, while other variable exons are not used. Each *Pcdh* variable exon has an exon-specific promoter, which contains a conserved sequence motif [39]. Fig. 5a shows the DNA methylation profile of *Pcdh-γ* clusters in each of the tissue types assayed in our study. The first variable exon of *Pcdh*-*α*and -*γ* was commonly methylated during lineage commitment from ES cells (Fig. 5a and Fig. S4c). *Pcdh-β* was also methylated, as was the first variable exon of *Pcdh-α* and -*γ* (Fig. S4a). These methylated regions were partially methylated in neural tissues. Previous reports showed that the upstream promoter of the first variable exons of the *Pcdh-α* cluster were methylated and that this methylation suppressed the expression of each *Pcdh-α* isoform [40]. Bisulfite sequence analysis of the protocadherin gene promoters showed that each germ layer similarly displayed mosaic methylation patterns in somatic lineages (Fig. 5b and Fig. S4b, d). Pcdhs are expressed predominantly in the nervous system (Fig. 5c, and data not shown). DNA methylation was not associated with *protocadherin* expression patterns among germ layers. These results suggest that DNA methylation regulates the expression of the first exon of *Pcdhs* in each cell.

**Figure 6:**
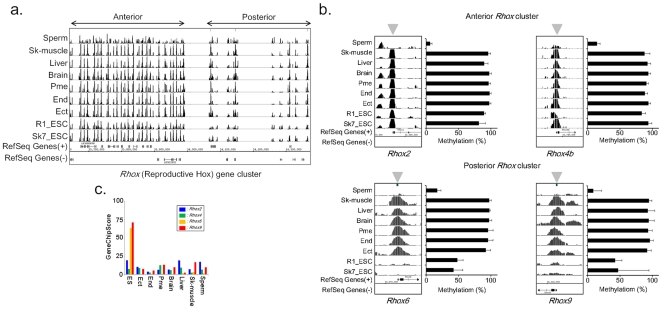
The *Rhox* cluster is classified into two regulated regions by DNA methylation. a) Overview of the DNA methylation profile of the *Rhox* cluster. b) The DNA methylation status of anterior and posterior *Rhox* genes. The left panel represents the MeDIP signal in each *Rhox* promoter. The right panel shows the quantitative estimation of DNA methylation by MALDI/TOFMAS. The arrowhead indicates the region of DNA analyzed for methylation. c) Expression profile of anterior and posterior *Rhox* genes.

The reproductive homeobox X-linked (*Rhox*) gene family was recently described in mice [41]. It is composed of 32 members that are all expressed in multiple reproductive tissues and placenta [42], [43]. *Rhox* genes are further divided into three subclusters: α, β, and γ based on proximity, expression patterns and sequence identity. It was previously reported that this cluster region is differentially methylated in a lineage-dependent manner [44]. Oda et al showed that this cluster region was hypomethylated in pre-implantation embryos and extra-embryonic tissues, but methylated during post-implantation development in the ICM/epiblast lineage and ES cells. However, we found that this cluster was divided into two classes based on the DNA methylation pattern. The anterior cluster (*Rhox*1-5) was methylated in all samples except sperm, and the posterior cluster was methylated after somatic differentiation (Fig. 6a). The posterior cluster was expressed in ES cells, but the anterior cluster was not (Fig. 6a, b, c), indicating that there is temporal regulation of *Rhox* gene expression by promoter methylation.

### Relationship between DNA methylation and histone methylation

**Figure 7:**
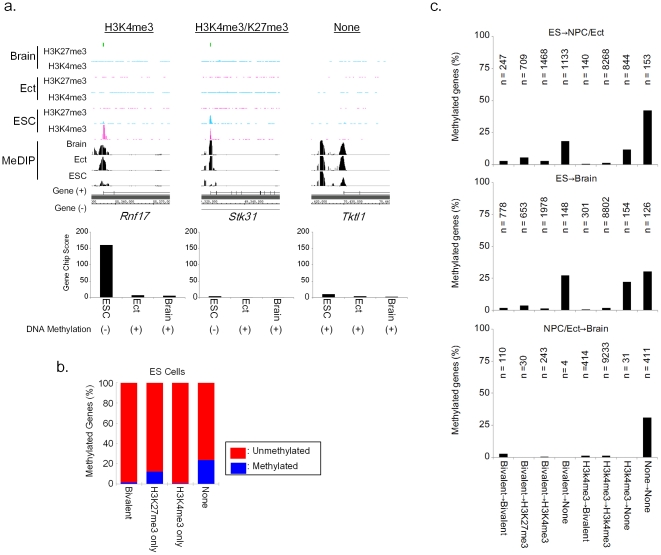
The exclusive relationship between DNA methylation and histone methylation. a) Representation of DNA methylation, histone methylation and gene expression. b) Percentage of genes DNA methylated with each histone modification. c) The percentage of DNA methylation in promoters is conditional on the histone methylation state in ES (Sk7_ESC) and NPC/Ect cells and in brain (n, number of promoters).

It is known that histone modification in promoter regions is associated with chromatin structure and gene expression. To understand how DNA methylation is regulated in these regions, we compared DNA methylation and histone modifications in promoter regions. H3K4me3 is a specific type of DNA methylation mark that is carried out by trithorax proteins that promote gene activation, and is located in the proximal regions of a TSS [45], [46]. H3K27me3 is a specific type of DNA methylation mark that is carried out by polycomb proteins that promote gene silencing, and is also located in the proximal regions of a TSS [45], [46]. We compared the DNA methylation profiles of ES cells, ectoderm and brain to those of a recently reported whole-genome histone map in ES cells, NPCs and whole brain [15], [18]. The histone marker patterns in the promoter regions were divided into four groups; (1) H3K4me3 or (2) H3K27me3 alone, (3) bivalent modification with H3K4me3 and H3K27me3, or (4) neither of these marks. We observed that these histone modifications had a mutually exclusive relationship with promoter DNA methylation status (Fig. 7a). In each sample, DNA methylation of proximal gene promoters was found to be at significantly lower levels in the presence of the H3K4me3 mark compared to the other marker patterns (Fig. 7b). These results suggest that DNA methylation and the presence of the H3K4me3 mark are mutually exclusive in ES cells.

We next analyzed how DNA methylation patterns change when ES cells differentiate into ectoderm *in vitro* (Table 5). We found that although most promoters remain unmethylated after *in vitro* differentiation, loss of H3K4me3 is correlated with gain of DNA hypermethylation (Fig. 7c). A similar trend was also observed when ES cells were compared with brain tissue, but not when NPCs were compared with brain. These results indicate the exclusive relationship between DNA methylation and H3K4 trimethylation during development, and show that this epigenetic conversion is observed at promoter regions of germ cell-specific genes in early development.

**Table 5:** The alternation of Histone modification during differentiation.

	ES→NPC		ES→Brain		NPC→Brain	
	Total	Me(+)	Total	Me(+)	Total	Me(+)
Bivalent→Bivalent*	247	7	778	12	110	3
Bivalent→H3K27me3*	709	41	653	23	30	0
Bivalent→H3K4me3*	1468	40	1978	26	243	1
Bivalent→None*	1133	209	148	40	4	0
H3K27me3→Bivalent	0	0	15	3	229	1
H3K27me3→H3K27me3	19	3	35	10	298	15
H3K27me3→H3K4me3	15	0	23	0	289	7
H3K27me3→None	72	27	33	18	15	2
H3K4me3→Bivalent*	140	1	301	2	414	5
H3K4me3→H3K27me3	102	9	97	7	75	0
H3K4me3→H3K4me3*	8268	126	8802	132	9233	124
H3K4me3→None*	844	101	154	34	31	0
None→Bivalent	0	0	1	1	342	8
None→H3K27me3	1	1	11	4	393	36
None→H3K4me3	2	0	18	0	1056	26
None→None*	153	65	126	38	411	128

*: analyzed histone alternations, ESC: Sk7_ESC, Number: number of Refseq genes

## Discussion

The tight control of gene expression programs at a given developmental stage is crucial for the governing of cell function and identity. The balance of stability versus plasticity in transcriptional programs represents an inherent regulatory mechanism for organ development. DNA sequence specific transcription factors are the most important mechanism for regulating expression or repression of a particular gene [12], [47]. However, evidence supports the concept that chromatin-based regulatory mechanisms, in addition to transcription factors, have important roles in establishing and maintaining transcriptional programs [12], [47]. Such regulation is comprised of DNA methylation, post-translational modification of DNA-bound histones and chromatin remodeling. DNA methylation is an efficient epigenetic repression mechanism in vertebrates. Embryonic lethality by ablation of Dnmts suggests that DNA methylation is essential for embryogenesis and cell differentiation. In this report, we performed DNA methylation profiling of early developmental stages; ES cells and the three early germ layers derived from ES cells, as well as of four terminally differentiated adult tissues. Our findings are summarized as follows. First, during cellular differentiation from ES cells into the three early germ layers, *de novo* methylation of target gene sets in gene promoter regions are common, with concordance rates reaching 67.3%. This figure represents a statistically significant enrichment in germ-cell specific genes. This result suggests that *de novo* methylation in promoter regions has a critical role during the early stage of embryogenesis. On the other hand, most of these genes were unmethylated in ES cells and in sperm (Fig. 3a). This observation may suggest that promoter regions of sperm and ES cells are epigenetically reprogrammed. *In vitro* differentiated germ layers have more promoter methylation than primary somatic tissues. Even if cultured cells acquire additional methylation under non-physiological conditions, these results explain why some genes are demethylated during terminal differentiation, as reported in a well-designed analysis of neurogenesis [13], [15]. Contrary to the hypothesis that additive CpG island methylation may be strongly associated with lineage restriction, lineage specific differences in gene methylation between the three early germ layers were extremely limited, as shown in Fig. 4a. Comprehensive developmental epigenomic studies have revealed that fine modulation of histone marking of key transcription factor binding sites have critical roles in regulatory networks. Notably, bivalent histone modification is specialized for fine regulation in a spatio-temporal manner. This modification may represent a useful chemical reaction system in response to environmental stimuli, allowing modulation of the state of chromatin for subsequent cellular adaptation. On the other hand, DNA methylation provides a chemically stable mark for mediation of long-lasting repression. These observations make it possible to understand how germ cell-specific gene-based mechanisms for silencing in the initial stages of reproductive cell fate determination evolved.

Second, most of the well-known imprinted loci are clearly detected across all samples as highly methylated regions. Our methylation profiling showed stable propagation of dense methylation from ES cells to differentiated cells. With respect to the *Igf2r* region, we could confirm specific reversal of imprinting and biallelic expression in ES cells, ectoderm and brain tissue, consistent with a previous report [38]. This finding indicates that DNA methylation is a fundamental mechanism for genome imprinting in somatic cells.

Third, we also identified specific roles for DNA methylation in the regulation of two cluster regions. It was observed that each promoter in the *Rhox* and *Pcdh* clusters was commonly methylated within a certain chromosomal range rather than individually methylated. The expression of these cluster-type genes was uniquely regulated. In fact, the first exons of the genes in the *Pcdh*-*α* and -*γ* clusters display individual expression patterns across different cell types, and each promoter alongside each first exon is regulated by a locus control region (LCR), which is a cis-regulatory sequence located in proximal regions of a constant exon. Therefore, these methylations at each first exon may determine the appropriate response to each LCR. On the other hand, the *Rhox* gene cluster showed two patterns of DNA methylation. Anterior *Rhox* genes, including *Rhox*1-5, were constitutively methylated except in sperm, but posterior *Rhox* genes, including *Rhox*6-12, remained unmethylated in ES cells and sperm. It has been shown that this gene cluster is preferentially expressed in reproductive organs and placenta [41]. These genes are important for reproductive organs, but anterior *Rhox* are expressed at a later point of postnatal testis development [41]. However, the *Rhox*6 and 9 posterior *Rhox* genes are expressed at an early point in postnatal testis development, but are not expressed in the testis [41]. Therefore, posterior *Rhox* genes might be important for ES cells themselves, or for the commitment of ES cells to adoption of a fate towards a reproductive organ.

In addition to *Rhox* genes, germ line-specific genes were enriched in commonly methylated genes, and associated with their expression. These genes were subdivided into three classes based on epigenetic and transcriptional status. The first gene class is methylated and not expressed in ES cells. The second gene class is not methylated and not expressed in ES cells. This second gene class showed bivalent histone marking and was methylated in somatic differentiation. The third gene class is not methylated and is expressed in ES cells. This third gene class showed only H3K4me3 histone marking and was also methylated in somatic differentiation. These findings suggest that DNA methylation is important for embryogenesis, but has little impact on the regulation of tissue-specific gene expression beyond reproductive tissue-associated gene expression. Interestingly, previous reports showed that many polycomb targets are highly enriched in developmental transcription factors, which are activated upon lineage commitment [48], [49]. Polycomb-mediated repression can be overcome by differentiation stimuli, whereas non-induced polycomb targets maintain H3K27me3 marking and polycomb occupancy [13]. Thus, it was suggested that stage-specific repression by polycomb functions ensure that further cell fate decisions are rigidly controlled. This theory suggests that DNA methylation engages in fate determination by fixing the suppressive state of genes. However, experimental evidence indicates that DNA methylation marks correlate with the loss of H3K4me3 marks, and previous reports showed that DNMT3 family members recognize the unmethylated lysine 4 (Lys 4) of histone H3 (H3K4me0) [50], [51]. It is known that Histone H3K4 tri-methylation is significantly enriched in high CpG promoter regions [18]. These finding suggest that H3K4me3 marks protect the promoter region from DNA methylation.

In this study, we estimated the DNA methylation pattern during early development by comparing the methylation profile of the three germ layers that differentiated from ES cells using an *in vitro* differentiation system. Our study was mainly restricted to CpG-rich regions. Genes in CpG-poor regions may also be regulated by DNA methylation. Indeed, most tissue-specific genes such as keratin and the olfactory receptor, are enriched in CpG-poor regions [52]. In fact, it is known that polycomb genes spatiotemporally regulate the expression of developmentally key transcription factors by histone methylation during embryogenesis [12], [13], [47]. Furthermore, polycomb-targets are also largely confined so that they associate with CpG-rich regions [53], [54]. A recent report shows that significant DNA methylation changes do occur in CpG-poor regions [14]. Therefore, further DNA methylation analysis focusing on CpG-poor regions is needed for a comprehensive understanding of the role of DNA methylation during development.

In conclusion, *de novo* methylation in promoter regions has a critical role in the establishment of long-lasting repression of germ cell-specific genes, which results in the restriction of cell fate towards non-germ line lineages.

## Supporting Information

Figure S1 **Gene expression profiles of ES cells and the three germ layers.** Representative genes down-regulated (blue) or up-regulated (red) after differentiation into specific cell lineages are shown.
(TIF)

Figure S2 **Methylation rate of CMR of various MATscores.** The methylation rate was calculated from the ratio of the number of methylated CpG against the number of all CpG sites in all sequenced clones. The average methylation rate is shown by open circles. N, number of analyzed CMR.
(TIF)

Figure S3 **Differentiation-coupled hypermethylation of promoters that regulate genes.** The left panel shows microarray detection of tissue-specific DNA methylation patterns. The right panel indicates the expression profile of DNA methylation-associated genes.
(TIF)

Figure S4 **The** ***Pcdh-α*** **and -** ***β*** **gene cluster is also methylated during differentiation into the three germ layers.** a) The DNA methylation status of the *Pcdh-β* cluster. b) Bisulfite sequence indicates the DNA methylation status of the *Pcdh-β4* promoter. c) The DNA methylation status of the *Pcdh-α* cluster. d) Bisulfite sequencing indicates the DNA methylation status of the *Pcdh-α4* promoter. The arrowhead indicates the bisulfite sequencing locus.
(TIF)

Table S1 Primer sequences used in this study.
(XLS)

Table S2 Gene Ontology (GO) terms associated with commonly methylated promoters in this study.
(XLS)
