# Supplementary figures and images for: DNA Methylation Profiling of Embryonic Stem Cell Differentiation into the Three Germ Layers
Source: PLoS One. 2011 Oct 7;6(10):e26052. doi: 10.1371/journal.pone.0026052 (PMC3189249; doi:10.1371/journal.pone.0026052)

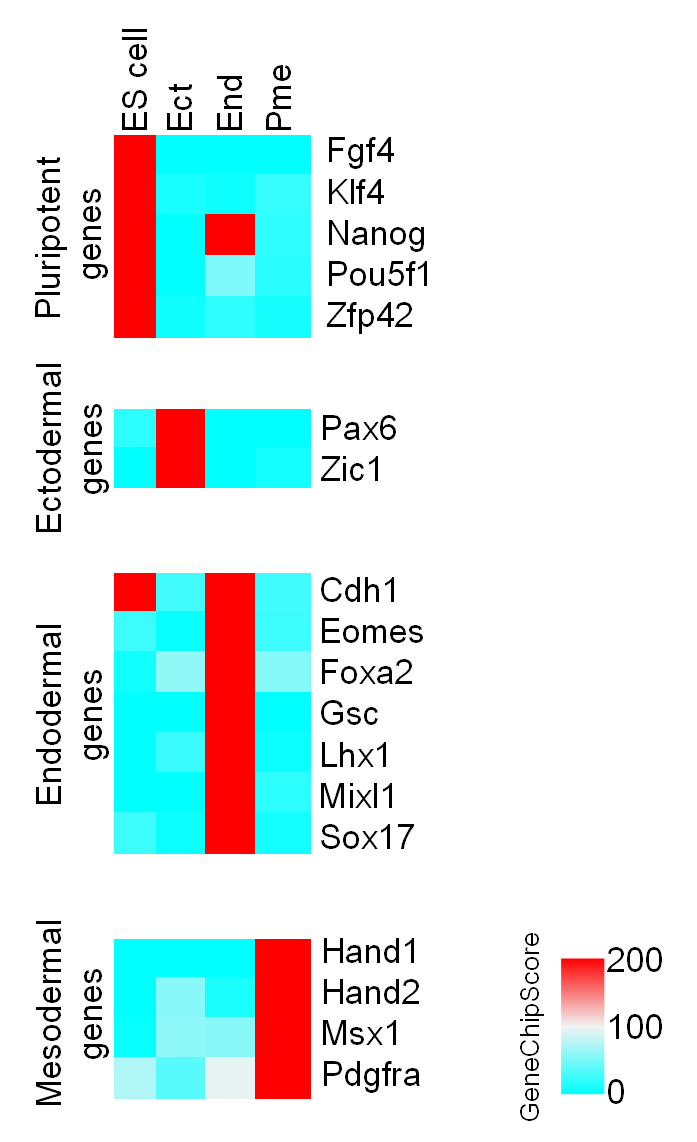

Supplement: Figure S1 — Gene expression profiles of ES cells and the three germ layers. Representative genes down-regulated (blue) or up-regulated (red) after differentiation into specific cell lineages are shown. (TIF) [file pone.0026052.s001.tif]

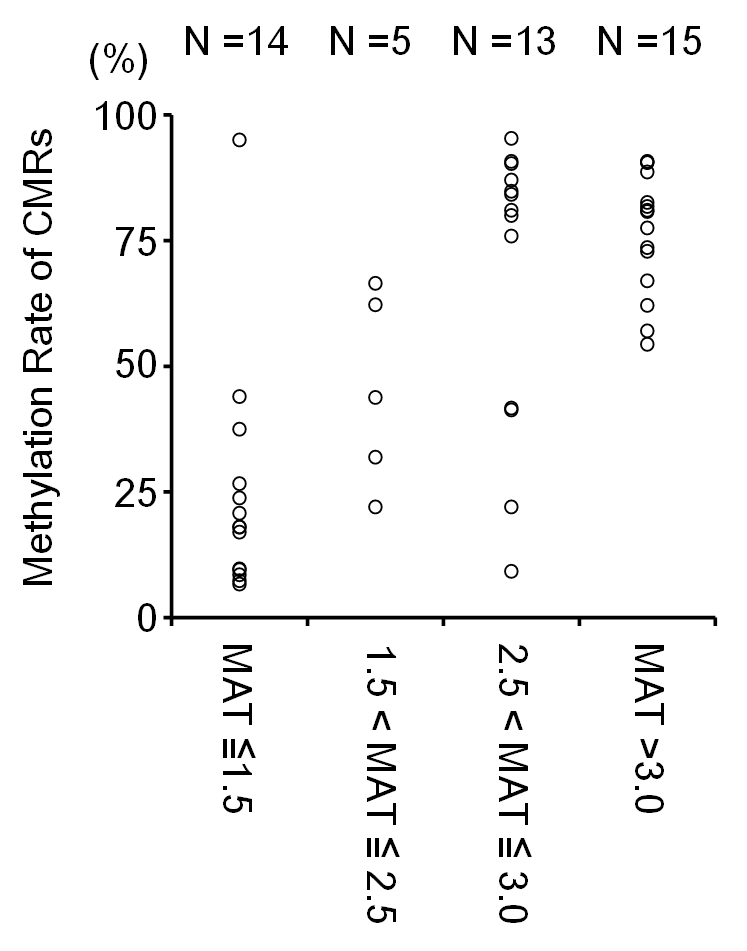

Supplement: Figure S2 — Methylation rate of CMR of various MATscores. The methylation rate was calculated from the ratio of the number of methylated CpG against the number of all CpG sites in all sequenced clones. The average methylation rate is shown by open circles. N, number of analyzed CMR. (TIF) [file pone.0026052.s002.tif]

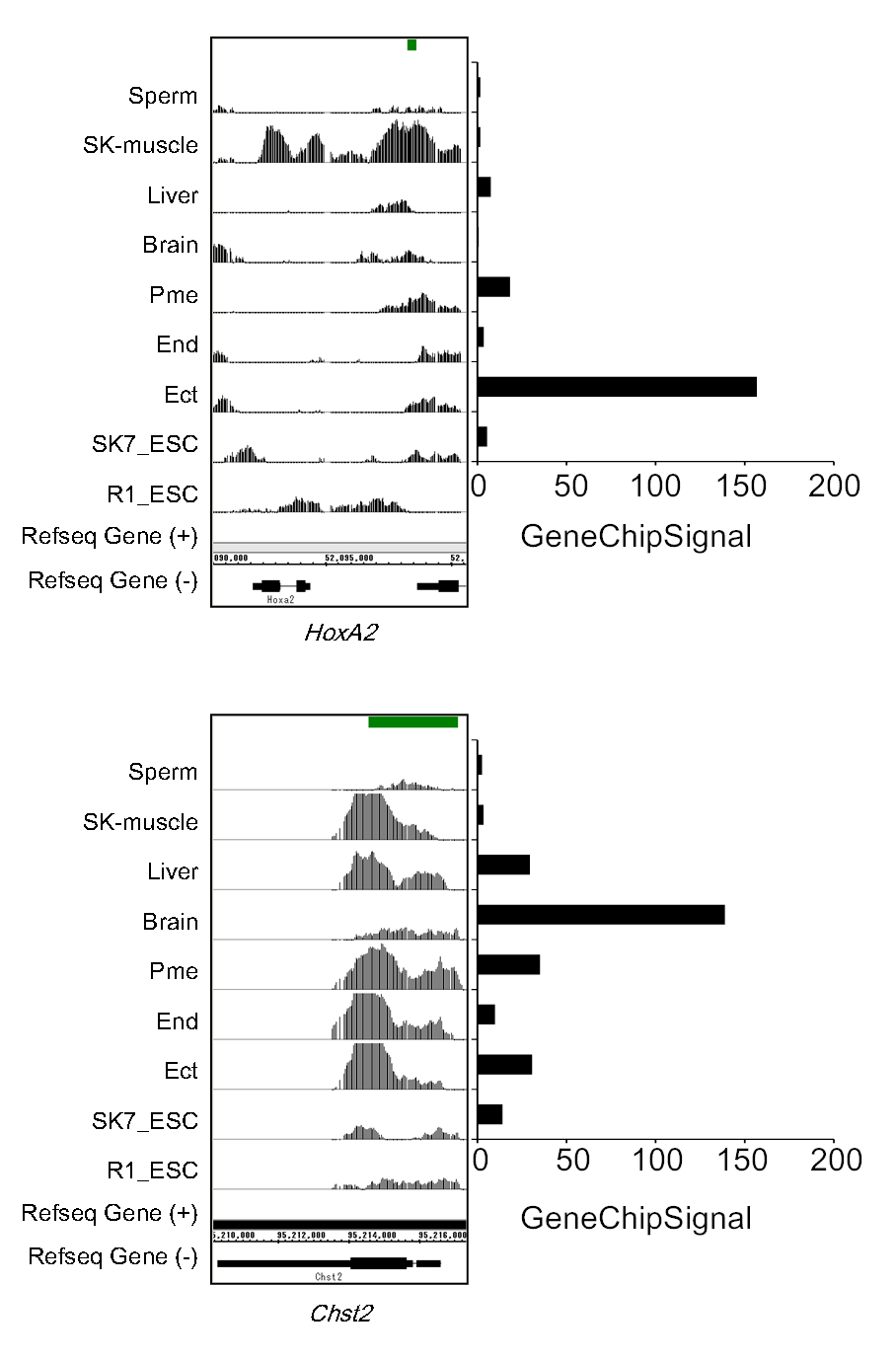

Supplement: Figure S3 — Differentiation-coupled hypermethylation of promoters that regulate genes. The left panel shows microarray detection of tissue-specific DNA methylation patterns. The right panel indicates the expression profile of DNA methylation-associated genes. (TIF) [file pone.0026052.s003.tif]

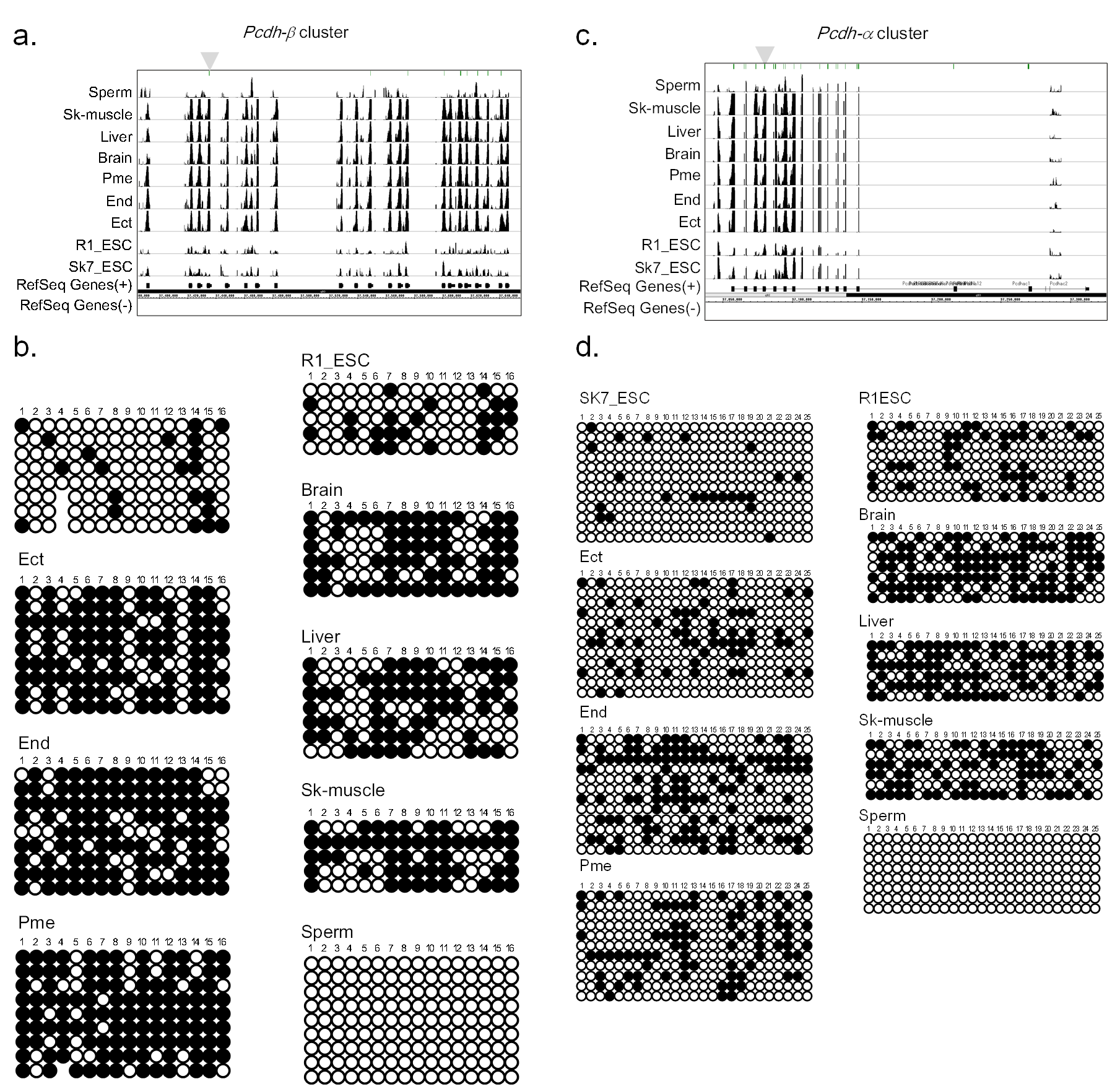

Supplement: Figure S4 — The Pcdh-α and - β gene cluster is also methylated during differentiation into the three germ layers. a) The DNA methylation status of the Pcdh-β cluster. b) Bisulfite sequence indicates the DNA methylation status of the Pcdh-β4 promoter. c) The DNA methylation status of the Pcdh-α cluster. d) Bisulfite sequencing indicates the DNA methylation status of the Pcdh-α4 promoter. The arrowhead indicates the bisulfite sequencing locus. (TIF) [file pone.0026052.s004.tif]
